# Characterization of Wear Properties of Pure Nickel Modified by Ni-Cr Composite and CaF_2_ Solid Lubricant Addition

**DOI:** 10.3390/ma15217511

**Published:** 2022-10-26

**Authors:** Mateusz Kotkowiak, Adam Piasecki

**Affiliations:** Institute of Materials Engineering, Faculty of Materials Engineering and Technical Physics, Poznan University of Technology, Jana Pawła II 24, 60-965 Poznan, Poland

**Keywords:** calcium fluoride, self-lubricating material, surface analysis, confocal microscopy

## Abstract

Nickel composites doped by chromium and calcium fluoride were produced by powder metallurgy. The friction coefficient of the samples containing 20% of the CaF_2_ was lower at elevated temperatures (600 °C) than the friction coefficient for the Ni-50%NiCr(80/20) composite (0.14 vs. 0.20). Sample surfaces were analyzed by the scanning electron microscope (SEM). EDS analysis proved tribofilm formation on the surface of the sample with CaF_2_ addition. A laser confocal microscope (LCM) was used to investigate the surface condition of the counter-sample after wear tests. The presence of the tribofilm reduced the wear of the frictional pair, and because of that the wear tracks were smooth. Tribofilm limited the abrasive wear and ploughing. Therefore, the tribofilm protected the sample and counter-sample from wear.

## 1. Introduction

Nowadays, nickel-based materials are very common because of their interesting properties such as high corrosion resistance. On the other hand, the main disadvantages of these materials are poor wear resistance and low hardness. Despite that fact, nickel-based materials have been widely used in different applications such as the automotive, shipbuilding, and aviation industries, as well as in nuclear reactors, which means they are exposed to high temperatures; hot corrosion; an aggressive environment; and wear at different, even very high, temperatures. Because of these difficult conditions, Ni-based materials have to be modified. Conventional Ni-based alloys could be modified by powder boriding [1,2], fluidized bed technology [3], paste boronization [4], or laser alloying [5,6]. Currently, ceramics materials based on nickel are very common, although they can be doped by other elements or chemical compounds. Nickel is often modified with additions of chromium, molybdenum, cobalt, or copper. Chromium is used to increase corrosion resistance at normal and elevated temperatures, as well as heat resistance. The addition of molybdenum increases the resistance to reducing acid. If copper is added to nickel, alloys will be characterized by high corrosion resistance in alkaline solutions, salts, and seawater. Moreover, Cobalt increases corrosion resistance at high temperatures [7,8]. One of the most frequently used nickel-based ceramics composites are Ni-Cr composite materials with varying additions of chromium or its compounds [9,10,11,12]. Additionally, because of their good properties, the Ni-Cr composites have been widely used as a coating material, to modify other materials, for example, stainless steel [13,14,15,16]. As Ni-Cr ceramic composites are so widely used, it is important to improve their wear properties, especially at high temperature. A possible way to strengthen the tribological properties of these materials is to modify them by adding solid lubricants. Solid lubricants have been divided into three groups, which differ in application temperature: −200 °C to room temperature (RT), and RT to 500 °C and above 500 °C [17,18]. Soft metals (Ag) [19,20,21], sulfides (MoS_2_, WS_2_) [22,23,24,25,26], and polymers (PTFE) [27] belong to moderate solid lubricant. Fluorides (BaF_2_, CaF_2_) [28,29,30,31], oxides (ZnO, PbO, and TiO_2_) [32,33], and salts (BaSO_4_, CaSO_4_) [34] form the group of high-temperature solid lubricants. The connection of Ni-based composite and self-lubricant, called self-lubricating materials, could be obtained by many methods: cold pressing and sintering [35], hot pressing and sintering [36], and spark-plasma sintering [37].

In this work, the Ni-based composites with the addition of NiCr(80/20) and CaF_2_ were investigated. Samples were produced by powder metallurgy. The first step was pressing by the hydraulic press followed by sintering carried out at 1000 °C for 2 h. Wear tests were conducted at room and at elevated temperatures, after tests samples were investigated by SEM and confocal microscopy.

## 2. Experimental Methods

### 2.1. Powder Metallurgy

The first step of the investigation was powder preparation. Powder particles are shown in the Supplementary Materials (Figure S1). Ni and Ni–Cr(80/20) powders are characterized by a spheroidal shape, whereas the particles of CaF_2_ have a cuboidal shape. The size of the particles did not exceed 10 µm (Figure S1a). Two powder mixtures were prepared: the first mixture contained 50 wt.% of pure Ni and 50 wt.% of NiCr(80/20), while the second mixture contained 40 wt.% of pure Ni, 40 wt.% of NiCr(80/20) and 20 wt.% of CaF_2_. The next step was pressing and sintering. Powder mixtures were pressed by the MP250M hydraulic press. The pressing pressure was equal to 11,952 kgf/cm^2^ (1.17 GPa). Sintering was conducted at 1200 °C in an inert atmosphere to avoid the oxidation of the samples. The heating rate was 250 °C/h, and so was the cooling rate [38].

### 2.2. Wear Tests

**Figure 1:**
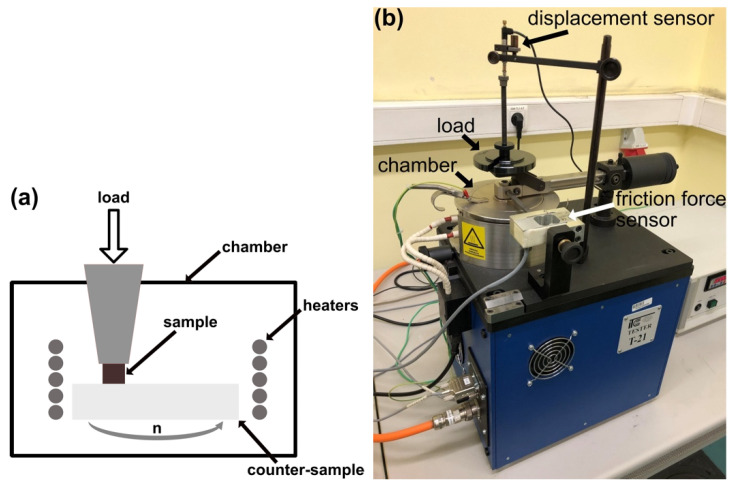
Pin-on-disc experiment: (**a**) scheme of the chamber, (**b**) T21 tribotester.

The pin-on-disc method was used to investigate the tribological properties of the composites. A detailed description of this method is described in the previous paper [32]. The parameters of the experiment were as follows: temperature (from room temperature (RT to 600 °C), time (1 h), the rotational speed of the counter-sample (120 min^−1^), and the load (4.9 N). The scheme and tribotester are shown in Figure 1.

### 2.3. Surface Analysis

Scanning electron microscope (SEM)—Tescan MIRA3 Brno, The Czech Republic—and optical microscope (OM)—Opta-Tech 40LAB, Warsaw, Poland—were used to investigate the surface conditions of the worn samples.

SEM investigations was conducted in contrast to the secondary electron (SE) and backscattered electrons (BSE). The BSE contrast is beneficial because it gives information about chemical composition diversity. The areas that contain light elements are dark, whereas heavy elements are bright. Additionally, sample surfaces were analyzed by the EDS method. The microanalyzer (Ultim^®^ Max 65, Oxford Instruments, High Wycombe, UK) was used to detect the elements appearing on the surface of the sample. Calcium, fluorine, nickel, chromium, and oxygen were analyzed. The results of the EDS analysis were shown in one color-element concentration map and in twelve color-scale maps. In this investigation, the accelerating voltage equal to 12 kV was used because the interaction volume has to be limited.

### 2.4. Confocal Microscopy

The confocal microscope (LSM 710, Carl Zeiss, Oberkochen, Germany), equipped with an HeNe laser with a wavelength of 543 nm, was used to investigate the surface of the samples after the wear test. Additionally, changes of roughness and the stereometrics profiles of samples were analyzed.

## 3. Results and Discussion

Composites were characterized by low porosity; additionally, the particles of CaF_2_ were evenly distributed in the matrix, which means the parameters of sample preparation were appropriate (Figure S2 in Supplement materials). Therefore, the wear test could be carried out.

**Figure 2:**
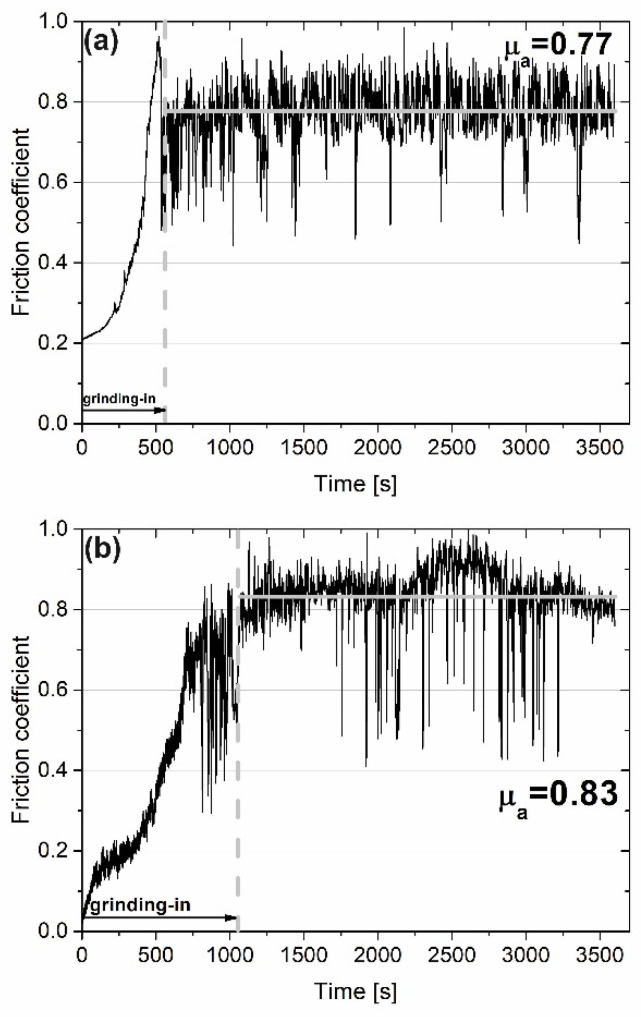
Results of wear test conducted at room temperature for sintered Ni-50%NiCr(80/20) (**a**) and Ni-40%NiCr(80/20)-20%CaF_2_ (**b**) (friction coefficient vs. time of friction).

**Figure 3:**
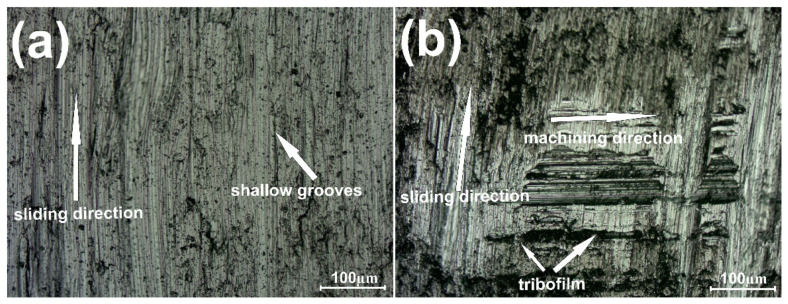
Surface condition of the counter-specimen mating with Ni-50%NiCr(80/20) (**a**) and Ni-40%NiCr(80/20)-20%CaF_2_ (**b**), after wear test conducted at room temperature.

Figure 2 shows the variation of friction coefficient (µ) for samples with and without the addition of the CaF_2_ at room temperature. The first stage of wear—grinding-in—lasted approximately 520 s for the Ni–50% NiCr composite and 1020 s for the sample with CaF_2_ addition. During the test conducted at RT, the average friction coefficient (µ_a_) for Ni–NiCr was lower than that for the self-lubricating composite (0.77 vs. 0.83). Figure 2 shows the surface of the counter-sample after the wear test. The surfaces were different: shallow grooves could be observed on the surface of the counter-specimen combined with composite without solid lubricant addition (Figure 3a). However, in Figure 3b, signs of previous machining were visible.

**Figure 4:**
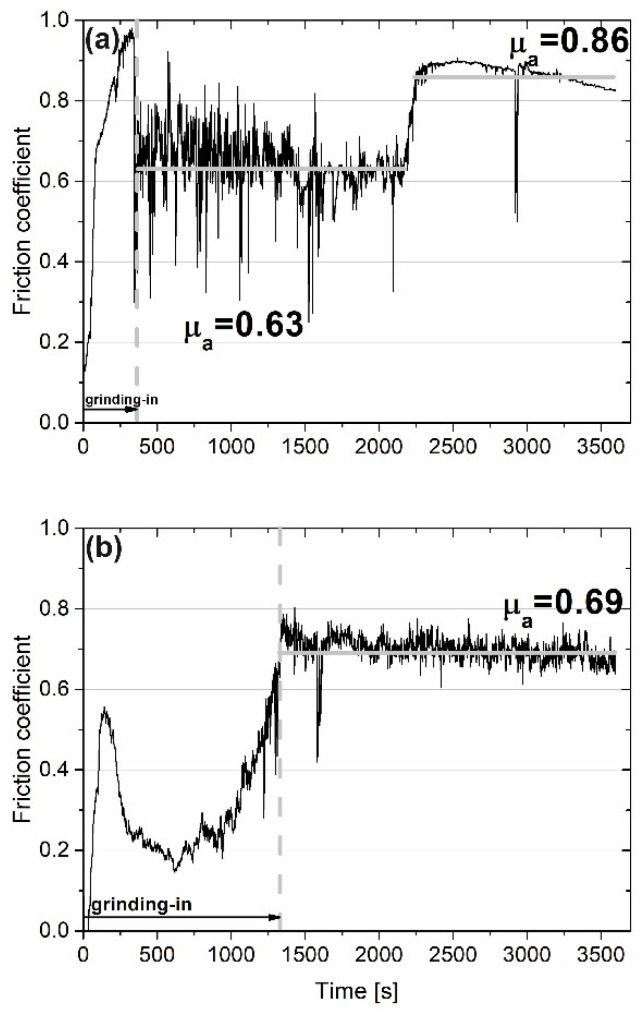
Results of wear test conducted at 200 °C for sintered Ni-50%NiCr(80/20) (**a**) and Ni-40%NiCr(80/20)-20%CaF_2_ (**b**) (friction coefficient vs. time of friction).

**Figure 5:**
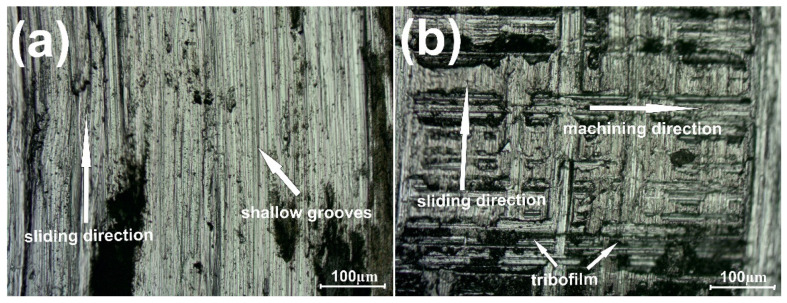
Surface condition of the counter-specimen mating with Ni-50%NiCr(80/20) (**a**) and Ni-40%NiCr(80/20)-20%CaF_2_ (**b**), after wear test conducted at 200 °C.

When the temperature of wear investigation increased to 200 °C, the course of the friction coefficient changed significantly (Figure 4). Grinding-in times were as follows: for the self-lubricating composite, approximately 1300 s; and for the composite without CaF_2_ addition, approximately 300 s. The µ_a_ value for the Ni–NiCr composite was 0.63, but after 2250 s it increased to 0.86. It may have been caused by adhesive wear, which meant that wear products were located between the mating parts. The µ_a_ for the sample with the addition of CaF_2_ was higher and equal to 0.69. Figure 5 shows the surface of the counter-specimens mated with samples. The signs of intensive abrasive wear were observed on the counter-specimen combined with the Ni–NiCr composite (Figure 5a). On the surface of the second counter-specimen, tribofilm was observed and, as a result, machining scratches were still visible after the wear test (Figure 5b).

**Figure 6:**
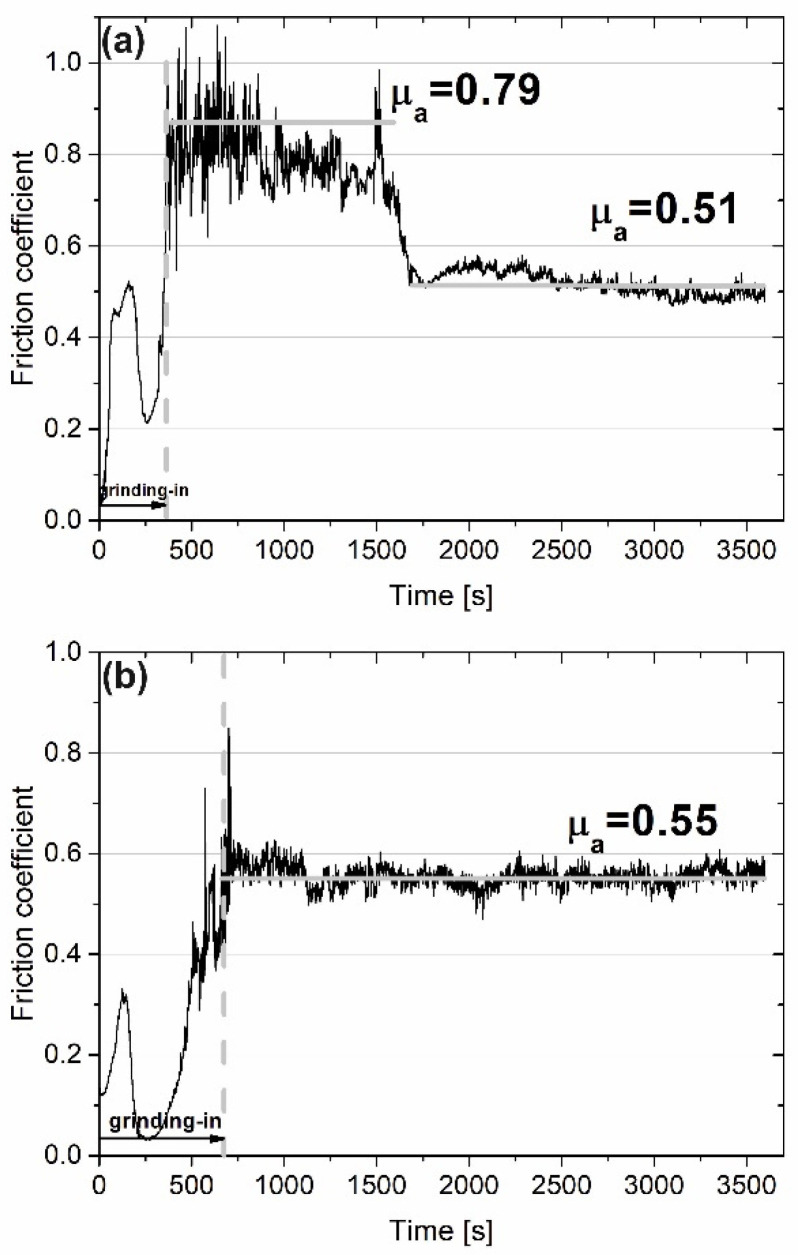
Results of wear test conducted at 400 °C for sintered Ni-50%NiCr(80/20) (**a**) and Ni-40%NiCr(80/20)-20%CaF_2_ (**b**) (friction coefficient vs. time of friction).

**Figure 7:**
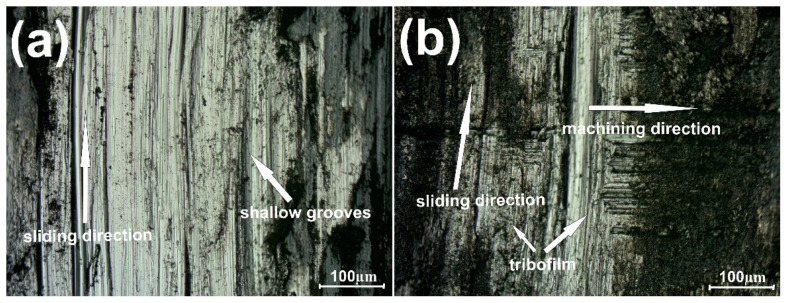
Surface condition of the counter-specimen mating with Ni-50%NiCr(80/20) (**a**) and Ni-40%NiCr(80/20)-20%CaF_2_ (**b**), after wear test conducted at 400 °C.

Figure 6 shows the variation of the friction coefficient during the wear test conducted at 400 °C. The grinding-in stage lasted approximately 300 s for the Ni–NiCr composite. After this time, the µ_a_ value was equal to 0.79 because of adhesive wear. However, after 1600 s, the average value of friction coefficient dropped drastically to 0.51. In the case of the self-lubricating composite, the first stage of wear—grinding-in—lasted approximately 700 s. After the grinding-in, the friction coefficient was stable over time and the µa was equal to 0.55. Differences between surfaces of counter-specimens mated with the Ni–NiCr composite and the self-lubricating composite are shown in Figure 7. Shallow grooves can be seen in Figure 7a, whereas tribofilm and previous machining signs are visible in Figure 7b.

**Figure 8:**
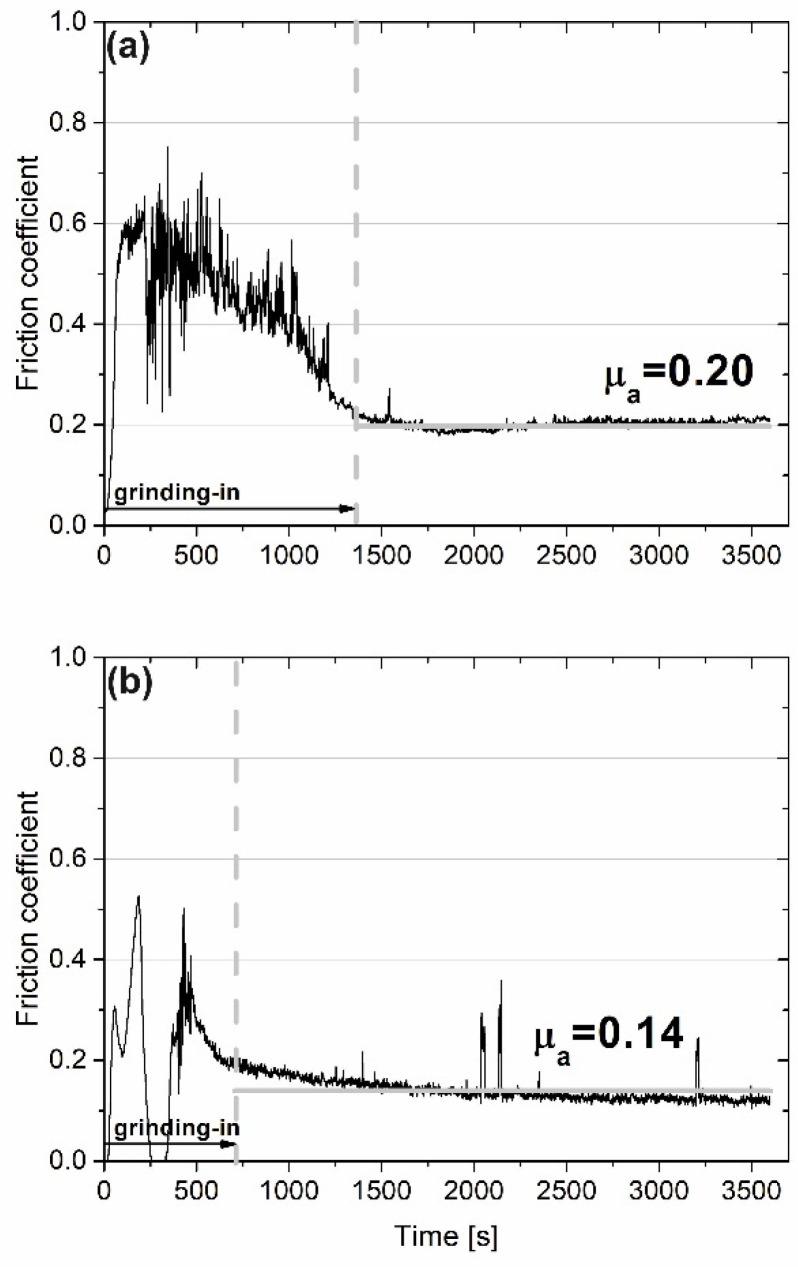
Results of wear test conducted at 600 °C for sintered Ni-50%NiCr(80/20) (**a**) and Ni-40%NiCr(80/20)-20%CaF_2_ (**b**) (friction coefficient vs. time of friction).

**Figure 9:**
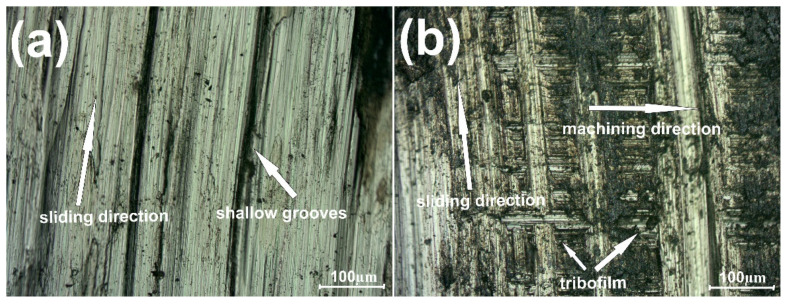
Surface condition of the counter-specimen mating with Ni-50%NiCr(80/20) (**a**) and Ni-40%NiCr(80/20)-20%CaF_2_ (**b**), after wear test conducted at 600 °C.

The results of wear investigation conducted at 600 °C are shown in Figure 8. At this temperature, the situation was different in comparison with RT, 200 °C and 400 °C. The grinding-in stage lasted longer for the sample without the addition of CaF_2_ (1350 s vs. 750 s). Additionally, the average friction coefficient (µ_a_) was lower for the sample with CaF_2_ addition (0.14 vs. 0.20). It formed because CaF_2_ is a high-temperature solid lubricant, and it has good lubrication properties above 500 °C. On the surface of the counter-sample combined with the Ni–NiCr composite, signs of intensive abrasive wear, i.e., shallow grooves, were visible (Figure 9a). However, on the counter-sample mated with self-lubricating, the composite traces of previous machining could be observed as well as tribofilm, which was produced during wear (Figure 9b).

Higher values of average friction coefficient and grinding-in times at room temperature, 200 °C, and 400 °C may have been caused by the relatively low temperature of the test. CaF_2_ is well known as a high-temperature solid-lubricant, which obtains the best lubrication properties above 500 °C. Below that temperature, CaF_2_ is too stiff, and it could not be smeared along the slip planes.

**Figure 10:**
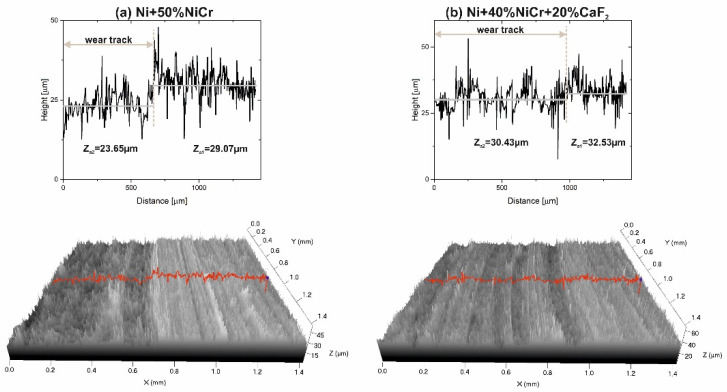
Stereometrics profiles of the counter-samples combined with Ni-50%NiCr(80/20) (**a**) and Ni-40%NiCr(80/20)-20%CaF_2_ (**b**), after wear test at room temperature.

**Figure 11:**
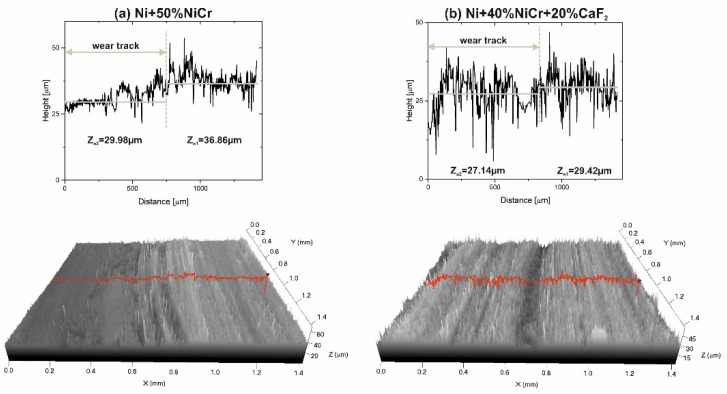
Stereometrics profiles of the counter-samples combined with Ni-50%NiCr(80/20) (**a**) and Ni-40%NiCr(80/20)-20%CaF_2_ (**b**), after wear test at 200 °C.

**Figure 12:**
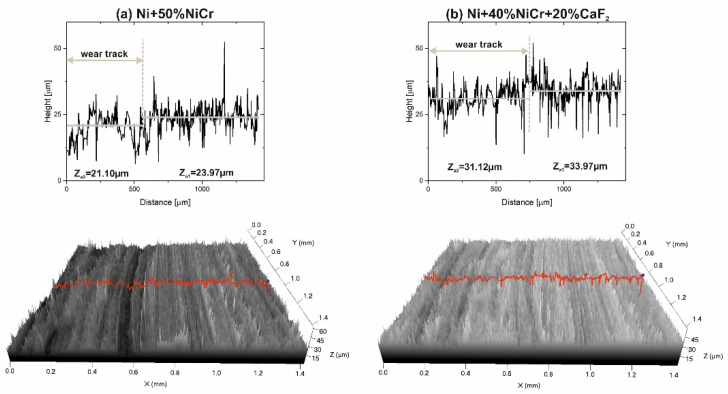
Stereometrics profiles of the counter-samples combined with Ni-50%NiCr(80/20) (**a**) and Ni-40%NiCr(80/20)-20%CaF_2_ (**b**), after wear test at 400 °C.

**Figure 13:**
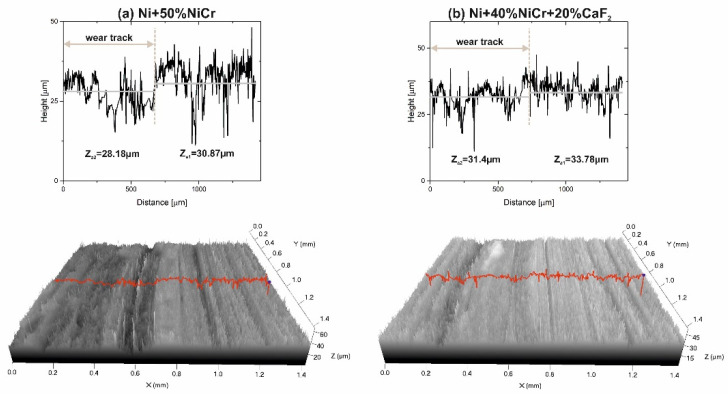
Stereometrics profiles of the counter-samples combined with Ni-50%NiCr(80/20) (**a**) and Ni-40%NiCr(80/20)-20%CaF_2_ (**b**), after wear test at 600 °C.

In Figure 10, Figure 11, Figure 12 and Figure 13, stereometrics profiles of counter-specimen surfaces and graphs with detailed changes in the height of counter-samples after wear test are shown. A comparison between the Ni–NiCr composite and the self-lubricating composite, tested at RT, is visible in Figure 10. It can be clearly seen that surface of the Ni–NiCr composites was rough, and the level of difference between the material and wear track (ΔZ) was greater than ΔZ for the self-lubricating composite (5.42 vs. 2.1). 

After the wear test conducted at 200 °C, the surface condition of the counter-sample mated with the Ni–NiCr composite was different (Figure 11a). The surface was smooth, but ΔZ value was very high, equal to 6.88. This may have been caused by adhesive wear, which meant that some parts of counter-sample were removed and stuck to sample surface. In the case of the counter-sample combined with the self-lubricating composite (Figure 11b), the profiles of the wear track and the base materials were similar and ΔZ was equal to 2.28. 

In Figure 12, the results of the investigation of the surfaces of the counter-samples tested at 400 °C are shown. On the stereometrics profile of the counter-sample mated with the Ni–NiCr composites, scratches were visible and the ΔZ value was equal to 2.87. If the counter-specimen was combined with self-lubricating composites, its surface looked different because only shallow grooves were observed (the ΔZ value was slightly lower (2.85 vs. 2.87)).

In Figure 13a, the surface condition of the counter-sample used with Ni–NiCr composite at 600 °C is shown. In this figure, numerous deep scratches were observed. The surface condition of the counter-samples mated with self-lubricating composites at 600 °C are visible in Figure 13b. The shallow grooves, as well as signs of adhesive wear, were observed on the surface of the counter-sample. ΔZ value for counter-sample combined with composite without CaF_2_ and with CaF_2_ were comparable and equal to 2.69 and 2.38, respectively. 

**Figure 14:**
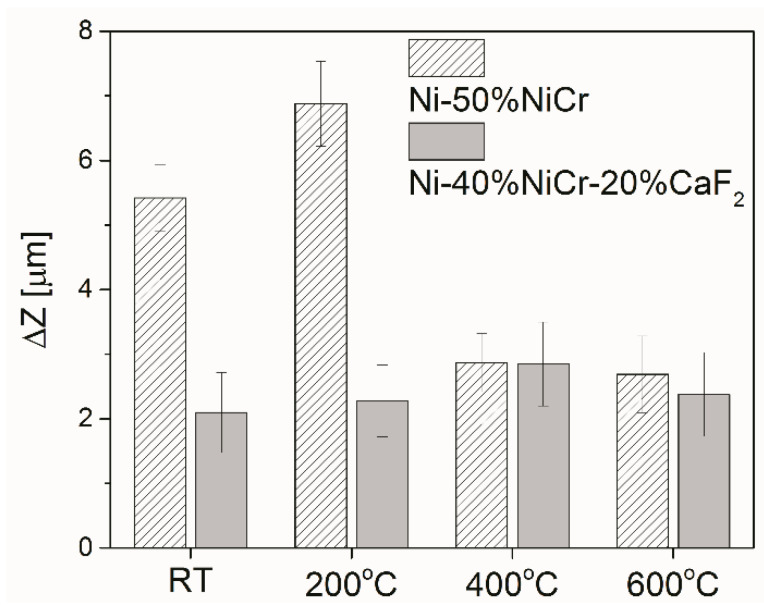
Level differences between the base material and wear track (ΔZ) of the counter-specimens.

A confocal microscopy investigation exhibited beneficial properties of tribofilm because it protected the sample and counter-sample against wear. The main wear mechanism for Ni–NiCr composites was abrasive wear, which was revealed by deep scratches; also, level differences (∆Z) between base material and wear track were higher (Figure 14). In the case of self-lubricating composites, some shallow grooves were observed on the surface.

**Figure 15:**
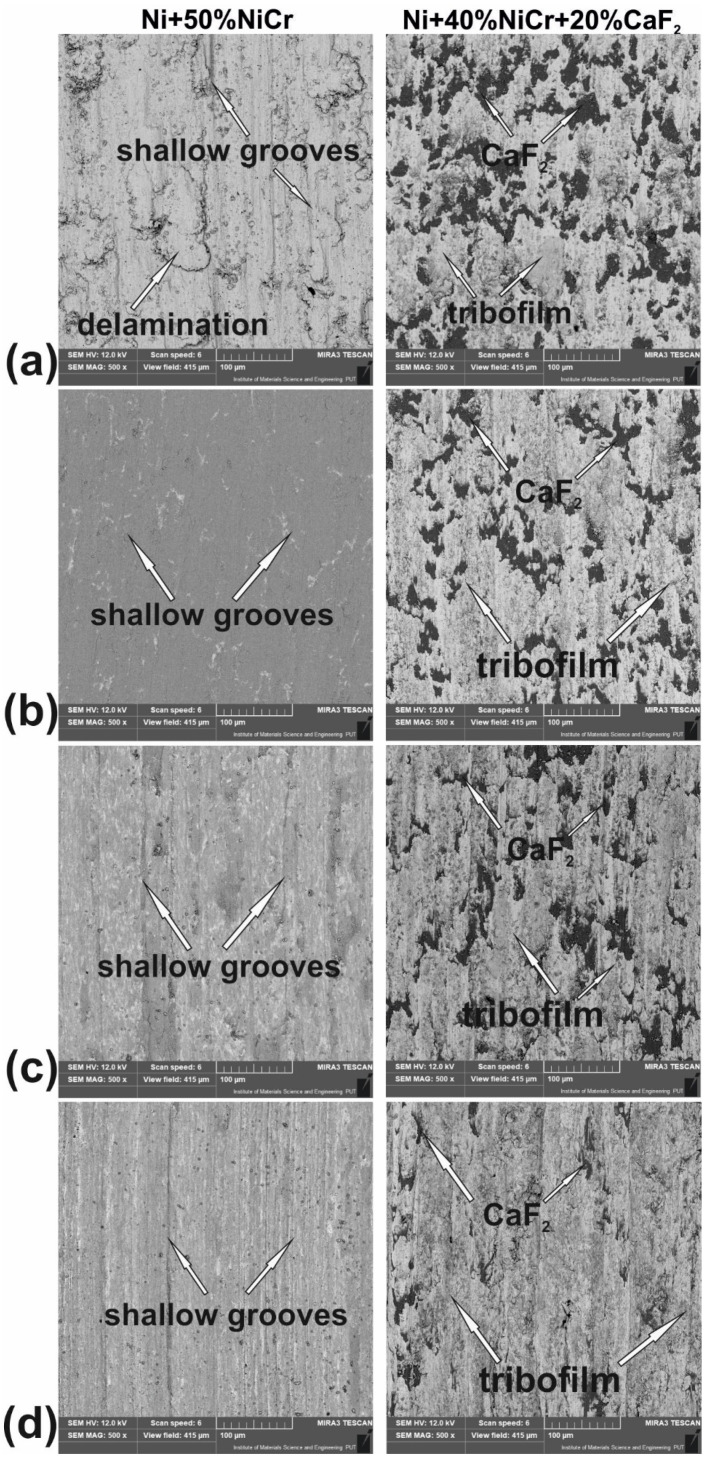
Surface condition of the sintered Ni-50%NiCr(80/20) and Ni-40%NiCr(80/20)-20%CaF_2_ after wear test at room temperature, (**a**) at 200 °C, (**b**) at 400 °C, (**c**) at 600 °C, and (**d**) (SEM).

**Figure 16:**
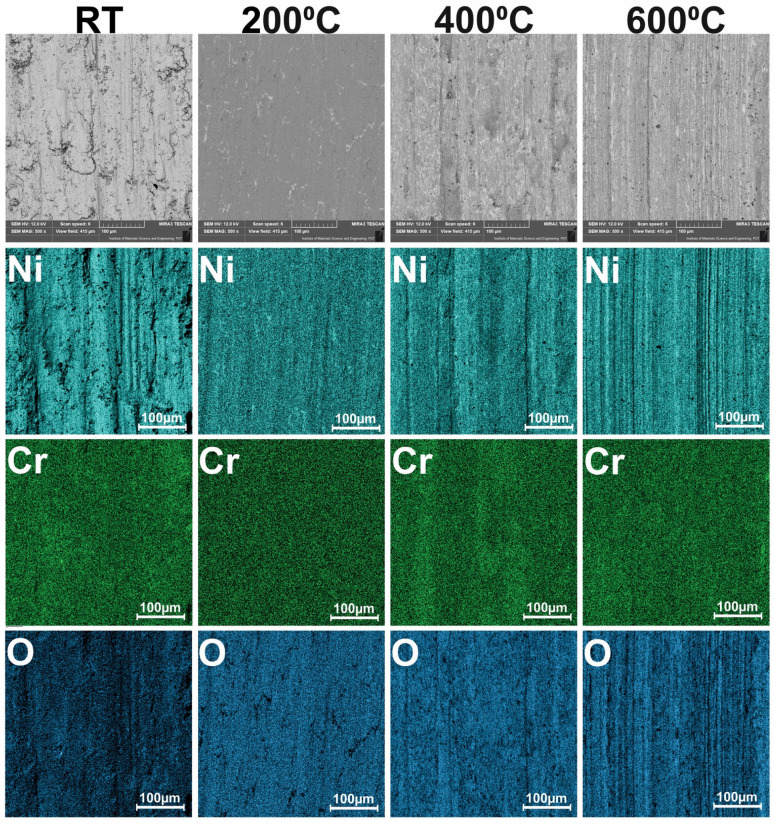
Results of EDS analysis of the worn Ni-50%NiCr(80/20) samples tested at different temperatures.

**Figure 17:**
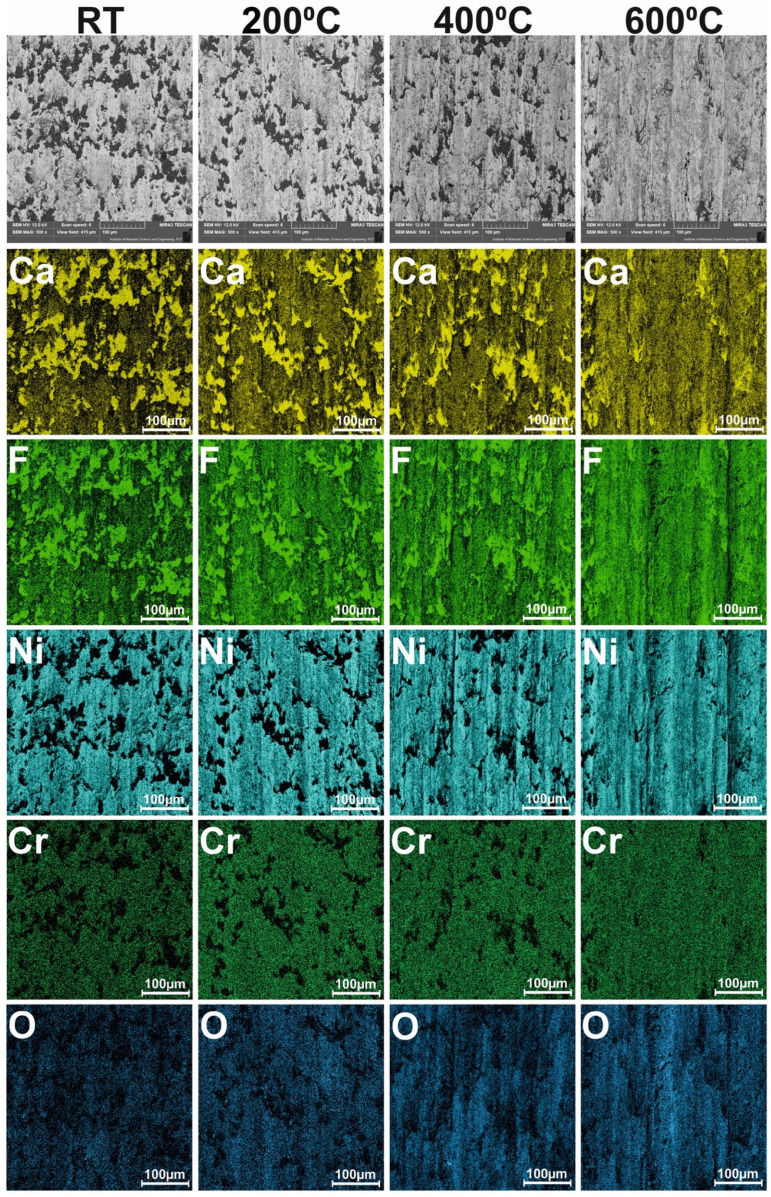
Results of EDS analysis of the worn Ni-40%NiCr(80/20)-20%CaF_2_ samples tested at different temperatures.

The surface conditions of samples and counter-samples after wear tests showed a great deal of information about the friction process and the wear mechanism. The surface conditions of the samples after the wear test conducted at different temperatures are shown in Figure 15. Shallow grooves were visible on the surface of Ni–NiCr composites, which appeared at all investigated temperatures. Additionally, at room temperature, delamination occurred. The surfaces of the self-lubricating composites were different. Tribofilm consisting of CaF_2_ was observed on sample surfaces, and tribofilm was characterized by varying thicknesses. The results of the EDS analysis are shown in Figure 16 and Figure 17. The following elements were investigated: Ni and Cr (the main elements of the samples), Ca and F (the main elements of solid lubricant), and O (to identify if oxidative wear occurred). In the case of Ni–NiCr (Figure 16) composite, the EDS analysis confirmed the presence of nickel and chromium. Additionally, increased oxygen content was observed on sample surfaces tested at 200 °C, 400 °C, and 600 °C. On the surfaces of the self-lubricating composites, an increased amount of Ca, F, Ni, and Cr was observed (Figure 17). Moreover, increased content of Ca and F occurred in places where the amount of Ni and Cr was reduced, because samples were covered by tribofilm. Increased content of O was observed on samples’ surfaces after a wear test conducted at elevated temperatures (200 °C, 400 °C, and 600 °C). Moreover, tribofilm protected samples against oxidation because the oxygen content was low in places where tribofilm formed.

**Figure 18:**
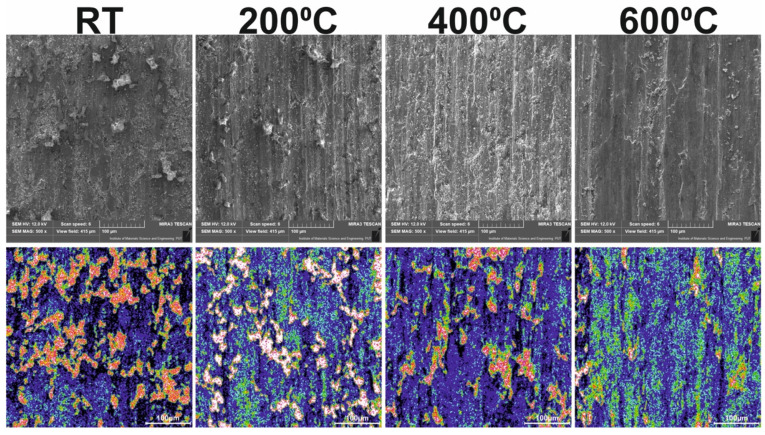
SEM and EDS images of the calcium concentration on the surface of the samples tested at different temperatures.

Figure 18 shows differences in the distribution of Ca on the surfaces of samples in 12-color scale. White areas contained a large amount of Ca, whereas in black areas, Ca was not detected. Thus, it was proved that tribofilm was characterized by diversified thickness. At room temperature and at 200 °C, there were more zones with brighter colors than at 400 °C and 600 °C. Differences emerged because CaF_2_ was not smeared very well on the surfaces of samples; therefore, numerous CaF_2_ clusters were observed. The situation changed at higher temperatures (400 °C and 600 °C). Then, CaF_2_ particles reached higher temperature and smeared very well on the surface of the sample. On the sample tested at 600 °C, numerous areas with similar intensity were observed, which meant that the tribofilm thickness was comparable.

**Figure 19:**
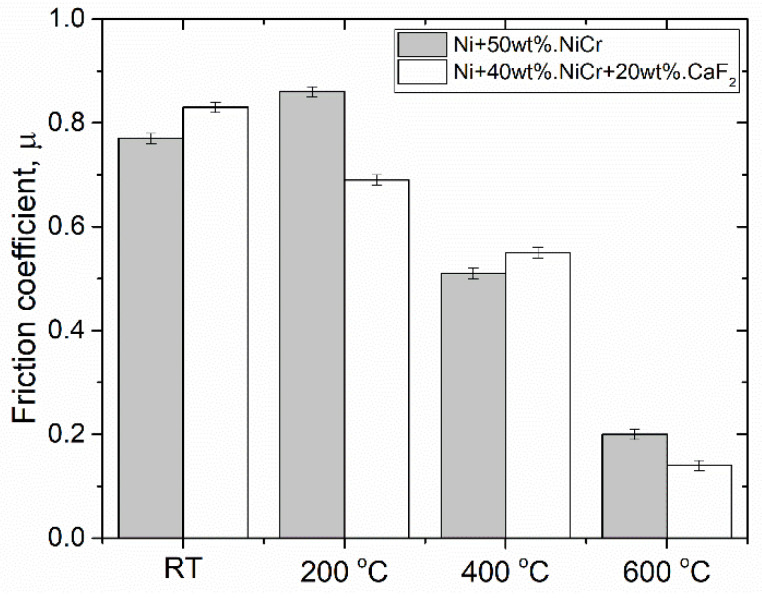
Average friction coefficient (µ_a_) of investigated materials.

It could be clearly seen that tribofilm protected samples against wear, thus producing lower values of the average friction coefficient (Table 1, Figure 19). The composites with the addition of CaF_2_ were characterized by lower values of µ_a_ at 200 °C and 600 °C, but on the other hand, at RT and 400 °C the µ_a_ were higher. When the values of µ_a_ were compared to the Figure 18, it could be clearly seen that on the surface of the composites tested at RT and 400 °C there were a lot of areas where CaF_2_ was not detected (black color), and this probably caused the higher values of µ_a_.

At 600 °C, the average friction coefficient was equal to 0.14 for the Ni-Cr composite with the addition of CaF_2_. The highest values of the coefficient of friction at 600 °C were observed for the Ni–Cr–Mo–Al composite with the addition of Ag and BaF_2_/CaF_2_ (approximately 0.3 in air and 0.16 in vacuum) [39]. Similar results of the friction coefficient were obtained in work [40]. Self-lubricating NiCr(80/20) composites with the addition of the following solid lubricants: Cr_2_O_3_ (20 wt.%), Ag (10 wt.%), and the eutectic mixture—BaF_2_/CaF_2_ (10 wt.%), were produced in work [41]. The lowest friction coefficients were at RT (~0.37), 200 °C (~0.33), and 400 °C (~0.32), which was probably caused by the addition of Ag, which is a moderate-temperature solid lubricant [17]. At 600 °C, the friction coefficient was equal to ~0.33, and it was two times higher than µ_a_ in this work. Additionally, the Ni alloy could be modified by the addition of 5% wt. CaF_2_ and 20 wt.% MoS_2_, and the friction coefficient, decreased form 0.41 at RT to 0.16 at 700 °C [42].

## 4. Conclusions

In this paper wear, the properties of the Ni–50%NiCr composite with and without the addition of CaF_2_ were investigated. The microstructure of the self-lubricating composites was beneficial because CaF_2_ particles were evenly distributed in the Ni–NiCr matrix.

1. At room temperature, the friction coefficient of the self-lubricating composite was higher than that of the composite without the addition of solid lubricant.
2. At 200 °C, initially, the µ_a_ value for the Ni–NiCr composite was lower than the value for the self-lubricating composite (0.63 vs. 0.69), but after some time, adhesive wear became the main wear mechanism and µ_a_ increased to 0.86.
3. At 400 °C, the µ_a_ value for the composite without the addition of CaF_2_,was equal to 0.79, and after 1500 s this value dropped to 0.51, whereas the average friction coefficient for the self-lubricating composite was equal to 0.55.
4. At 600 °C, the average friction coefficient was lower for the self-lubricating composite in relation to the Ni–NiCr composite (0.14 vs. 0.20).
5. Grinding-in times were lower for the sample without the addition of solid-lubricant at room temperature, 200 °C, and 400 °C. However, at 600 °C, the grinding-in time for the self-lubricating composite was approximately two times lower than for the Ni–NiCr composite (720 s vs. 1350 s).
6. A surface analysis of the samples proved that the self-lubricating composite was coated by the thin tribofilm layer. EDS analysis showed that tribofilm consisted of calcium and fluorine and was characterized by diversified thickness.
7. The EDS analysis confirmed that tribofilm was well smeared on the sample surface at higher temperatures (400 °C and 600 °C) and the tribofilm protected samples against oxidation.

## Figures and Tables

**Table 1:** Average friction coefficient (µ_a_) of investigated materials.

	RT	200 °C	400 °C	600 °C
	µ_a_			
Ni + 50 wt.% NiCr	0.77 ± 0.01	0.86 ± 0.01	0.51 ± 0.01	0.20 ± 0.01
Ni + 40 wt.% NiCr + 20 wt.% CaF_2_	0.83 ± 0.01	0.69 ± 0.01	0.55 ± 0.01	0.14 ± 0.01

## Data Availability

The data presented in this study are available on request from the corresponding author.

## Supplementary Materials

The following supporting information can be downloaded at: https://www.mdpi.com/article/10.3390/ma15217511/s1, Figure S1: The powders used in this investigation: pure Ni (a), Ni–Cr (80/20) (b) and CaF_2_ (c). Figure S2: Microstructure of produced sinters: Ni + Ni–Cr (a) and Ni + Ni–Cr + 20%CaF_2_ (b).
