# Supplementary material for: Characterization of Wear Properties of Pure Nickel Modified by Ni-Cr Composite and CaF2 Solid Lubricant Addition
Source: Materials (Basel). 2022 Oct 26;15(21):7511. doi: 10.3390/ma15217511 (PMC9659272; doi:10.3390/ma15217511)
Supplement: Supplementary file 1 [file materials-15-07511-s001.zip › materials-1955647-supplementary.pdf]

Supplementary material
for

Characterization of Wear Properties of Pure Nickel Modified by Ni-Cr Composite and CaF_2 Solid Lubricant Addition

Mateusz Kotkowiak * and Adam Piasecki *

Institute of Materials Engineering, Faculty of Materials Engineering and Technical Physics, Poznan University of Technology, Jana Pawła II 24, 60-965 Poznan, Poland
* Correspondence: mateusz.kotkowiak@put.poznan.pl (M.K.); adam.piasecki@put.poznan.pl (A.P.)

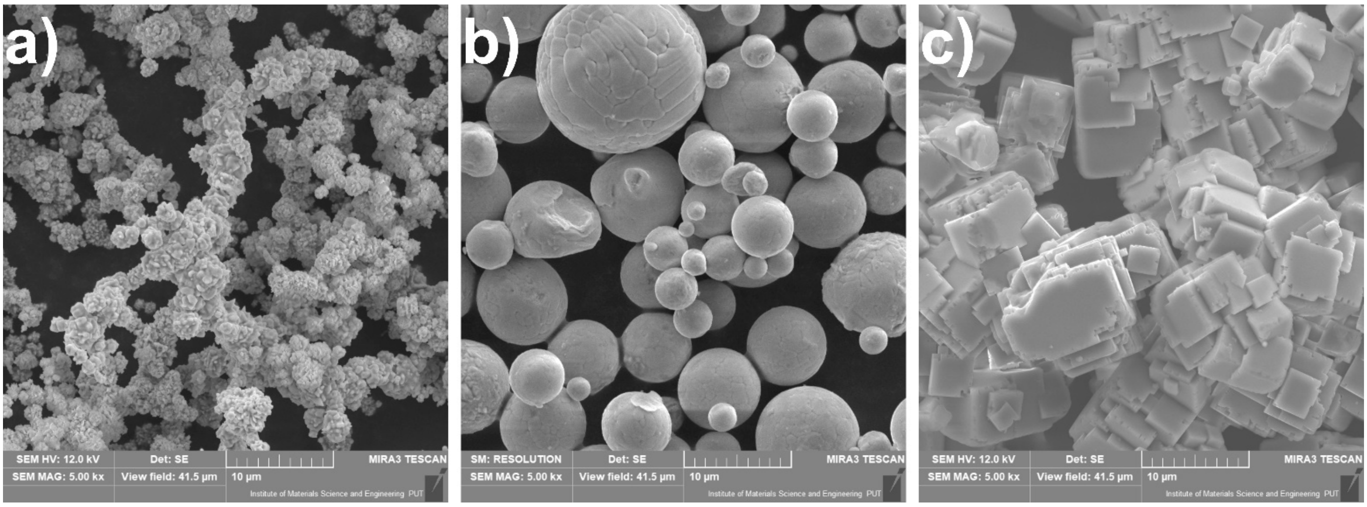

Figure S1. The powders used in this investigation: pure Ni (a), Ni–Cr (80/20) (b) and CaF_2 (c).

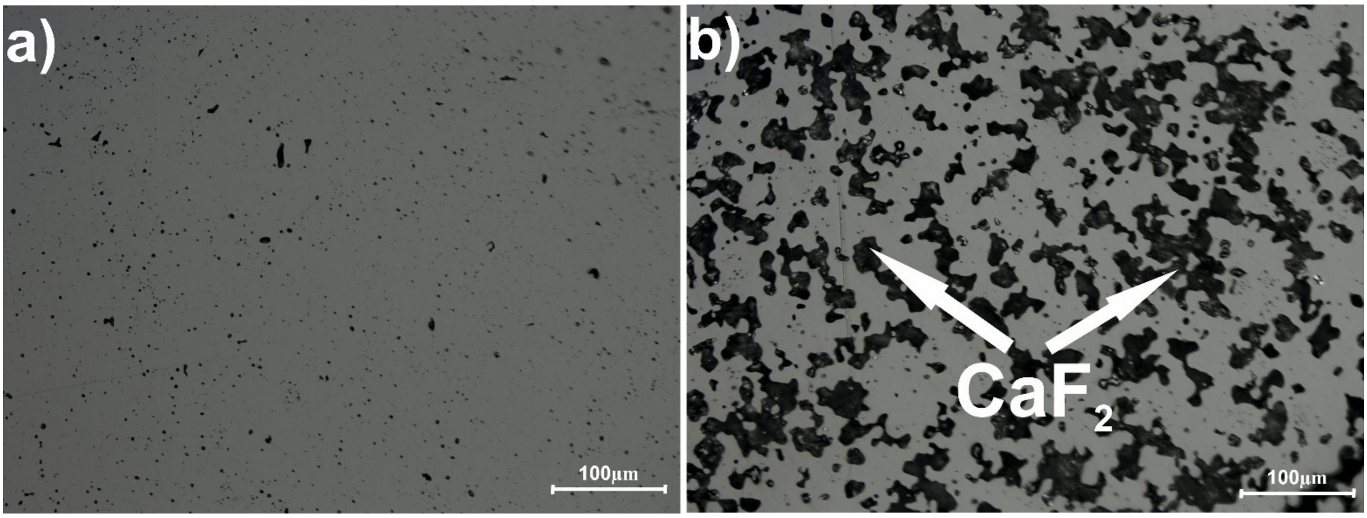

Figure S2. Microstructure of produced sinters: Ni + Ni–Cr (a) and Ni + Ni–Cr + 20%CaF_2 (b).
